# Almost a Fatal Accident from Anesthesia by Scopolamin, Morphin, and Chloroform

**Published:** 1905-12

**Authors:** 


### Almost a Fatal Accident from Anesthesia by Scopolamin, Morphin, and Chloroform.

M. Monod (*Journal de méd. de Paris,* September 24, 1905) reported the following case to the société de chirurgie, and declared that he had abandoned the scopolamin-morphin method of anesthesia. The patient, a young woman, 27 years of age, had been operated upon by a cholecystenterostomy, and besides the dose of scopolamine and morphine, had received a rather large quantity of chloroform. She was seized, near the close of the operation, with cyanosis, dyspnea, dilated pupils and weak pulse. Then the heart ceased beating and respiration stopped. After half an hour of artificial respiration and other measures, the patient revived, but for several days remained in a grave condition, with a weak heart. The reporter thought that the scopolamin-morphin injections were largely to blame.—*New York Medical Journal.*

